# High-Content Screening of Wind-Dampness Dispelling Traditional Chinese Medicines against Podocyte EMT Induced by IgA1

**DOI:** 10.2174/0113862073350741250410053000

**Published:** 2025-04-29

**Authors:** Jin Yu, Lingli Zhu, Yue Sun, Caifeng Zhu

**Affiliations:** 1 Department of Nephrology, Hangzhou Hospital of Traditional Chinese Medicine (Hangzhou TCM Hospital Affiliated to Zhejiang Chinese Medical University), Hangzhou, Zhejiang, P. R. China;; 2 Department of TCM, Jinhua Municipal Central Hospital, Jinhua, Zhejiang, P.R. China

**Keywords:** Immunoglobulin a nephropathy, podocyte injury, wind-dampness dispelling traditional chinese medicines, epithelial-to-mesenchymal transition, high-content screening, drug intervention

## Abstract

**Objective:**

Immunoglobulin A (IgA) Nephropathy (IgAN) is characterized by pIgA1 dep-osition in the glomerular mesangium, resulting in podocyte Epithelial-to-Mesenchymal Transi-tion (EMT). Traditional Chinese Medicines (TCMs) have been used for the treatment of IgAN for several years and have demonstrated positive efficacy. The present study aimed to establish a High-Content Screening (HCS) method for identifying wind-dampness dispelling TCM extracts that can mitigate podocyte EMT induced by pIgA1.

**Material and Methods:**

IgA1 from IgAN patients was used to establish a podocyte EMT model. The expression of EMT markers, including desmin, podocalyxin, and podocin, was assessed by Immunocytofluorescence (IF). An image-based HCS method was established to identify wind- dampness dispelling TCMs with the anti-EMT activity of podocyte EMT by automatic acquisition and processing of dual-fluorescent labeled images.

**Results:**

A total of 21 wind-dampness-dispelling TCM extracts were screened using the HCS system, leading to the identification of eight wind-dampness-dispelling TCM extracts exhibiting anti-EMT activity. These eight extracts inhibited the pIgA-induced expression of desmin while upregulating the expression of podocalyxin and podocin.

**Conclusion:**

By quantifying the changes in the expression of EMT markers during pIgA1- induced EMT, this study successfully identified wind-dampness dispelling TCM extracts with anti-EMT properties using an HCS system. In addition, the proposed approach presents a novel avenue for the identification of wind-dampness-dispelling TCMs for the treatment of pIgA1- induced EMT.

## 1 INTRODUCTION

Immunoglobulin A (IgA) Nephropathy (IgAN) is the most common type of primary glomerulonephritis worldwide and a leading contributor to Chronic Kidney Disease (CKD) and renal failure [1]. Podocyte wraps around glomerular capillaries and serve a crucial function in the glomerular filtration process [2]. Podocyte injury is a key factor in the progression of IgAN and triggers a series of changes. The common clinical outcome of podocyte injury includes but is not limited to proteinuria, which also leads to an increase in the mesangial extracellular matrix, kidney injury, glomerulosclerosis, and renal failure [3]. Epithelial-to-Mesenchymal Transition (EMT) emerges as a pivotal biological process that plays a central role in these pathophysiological processes. EMT is characterized by the transition of epithelial cells into mesenchymal-like cells, which leads to alterations in cell phenotypes and markers [4]. Decreased expression of mesenchymal markers and increased expression of podocyte markers (nephrin, podocin,p-cadherin, and ZO-1) resultsin podocyte EMT [5]. Emerging evidence has established that EMT is a significant mechanism that contributes to glomerulosclerosis in IgAN [6]. Consequently, the podocyte EMT is a crucial target for interventions in IgAN.

Chinese medicines, particularly wind-dampness-dispelling Traditional Chinese Medicines (TCMs), present advantages in the treatment of IgA nephropathy. Clinical practice has demonstrated the favorable therapeutic effects of wind-dampness dispelling TCMs in IgAN, significantly alleviating proteinuria and improving renal function in patients with IgAN [7]. Specifically, the effectiveness of *Tripterygium wilfordii Hook. f.* and *Stephaniae Tetrandrae Radix* in protecting podocytes and reducing proteinuria levels have been widely substantiated in the scientific literature [8, 9].

However, the diversity of wind-dampness dispelling TCMs suggests the importance of screening more TCMs targeting podocyte EMT for the prevention and treatment of IgAN. This was the focus of the present study. We used a High-Content Screening (HCS) approach to systematically evaluate a panel of wind-dampness-dispelling TCMs for their potential to inhibit podocyte EMT. HCS systems are advanced experimental platforms that combine cell biology, optics, and computer science to enable precise quantitative analysis of biological samples. They have broad applications in life science research, including drug screening, assessing cellular toxicity, creating disease models, and exploring drug mechanisms. In this study, we established a podocyte EMT model of IgA nephropathy *in vitro* and used a high-content analysis platform to screen and search for new wind-dampness-dispelling TCMs that can treat IgAN by preventing podocyte EMT. This study provides a new method for the development of novel drugs for IgAN.

## 2 MATERIAL AND METHODS

### 2.1 Subjects

Twenty newly diagnosed primary patients with IgAN were recruited at the Hangzhou Traditional Chinese Medicine Hospital based on renal biopsy results. The inclusion criteria included: (1) IgAN proven by kidney biopsy with glomerular mesangial IgA deposits on immunofluorescence and compatible lesions on light microscopy. However, cases of secondary IgAN, such as lupus nephritis and Henoch–Schönlein purpura, were not included. (2) Age between 16 and 65 years old; (3) No use of steroids, immunosuppressants, tripterygium glycosides, ACE inhibitors, or ARB drugs within the past 2 weeks. The exclusion criteria included (1) secondary IgAN, acute or chronic infections, liver cirrhosis, connective tissue disease, or other diseases that could cause abnormal IgA expression; (2) treatment with steroids, immunosuppressants, ACE inhibitors, or ARB drugs; (3) occurrence of mucosal reactions during blood collection. Each patient provided 10mL of blood and the samples were centrifuged at 3000 rpm for 15 min. The serum was isolated and frozen at -20°C until the isolation of IgA. All participants provided written informed consent, and the study was approved by the Hangzhou Traditional Chinese Medicine Hospital Ethics Committee (2021LH004).

### 2.2 Purification of Polymeric and Monomeric IgA1 using Jacalin Affinity Chromatography

Sera from patients with IgAN were diluted 1:1 with PBS and added to a column packed with jacalin agarose (Thermo Scientific, USA). Flow-through absorbance at 280 nm was monitored for each fraction to establish a baseline. The column was then washed with PBS until the baseline was reached to ensure that all IgA1 was bound to jacalin. Bound IgA1 was subsequently eluted using 0.1 M melibiose (Sigma, USA) in PBS. The eluted fractions were collected until a baseline was reached. The eluted fractions were combined, concentrated, and loaded on a Sephacryl S-200 molecular sieve column. Western blotting was performed to confirm IgA1 content in the samples. The purified IgA1 was incubated at 63°C for 150 min to obtain pIgA1. The concentration of pIgA1 was measured with μDrop plate (μDrop N12391, Thermo Scientific, USA) using a microplate reader (Varioskan LUX, Thermo Scientific, USA). Finally, the samples were stored at -80°C for future studies.

### 2.3 Cell Culture

SV40-transformed murine glomerular mesangial cells (SV40 MES13) were purchased from CellCook Inc. (Guangzhou, China) and maintained in a 3:1 mixture of DMEM/F-12 medium (CellCook) supplemented with 5% Fetal Bovine Serum (FBS, Gibco BRL, Grand Island, NJ, USA) at 37 °C in an atmosphere of 5% CO_2_.

Mouse Podocyte Clone 5 (MPC5) cells were obtained from Dr. Peter Mundel (Mount Sinai School of Medicine, New York, USA) and maintained in RPMI-1640 (Gibco BRL) supplemented with 10% FBS (Gibco BRL), and 100 U/mL interferon (IFN)-γ (PeproTech, Inc., Rocky Hill, NJ, USA) at 33°C with 5% CO^2^ for proliferation. Cell differentiation was induced by incubating in RPMI-1640 without IFN-γ at 37°C with 5% CO_2_ for 14 days.

### 2.4 Preparation of the Conditioned Medium

SV40 MES13 cells were stimulated with 1% FBS-RPMI1640 medium with different concentrations of pIgA (0, 25, 50, 100, and 150 µg/ml) for different durations (24 and 48 hours). The supernatant was collected and stored at -70°C for assay of the level of Tumor Necrosis Factor-alpha (TNF-α). TNF-α concentration was determined by ELISA. IF staining was used to assay the expression alterations of α-SMA, which were monitored using fluorescent microscopy. The stimulation conditions that induce an increase in TNF-α levels in the culture supernatant and expression of α-SMA in mesangial cells were determined as the optimal intervention conditions.

### 2.5 Induction of EMT in Podocytes

MPC5 cells were cultured in a six-well culture plate (2*10^6 cells/well) with different concentrations (0- to 8-fold diluted medium) of IgA-MES-conditioned medium for 48 hours. IF staining was used to assay the expression of desmin, podocalyxin, and podocin, which were monitored using fluorescence microscopy. The stimulation conditions that increased desmin expression and decreased podocalyxin and podocin expression in MPC5 cells were determined to be the optimal intervention conditions.

### 2.6 Validation of the Screening Method Based on the HCS System

MPCs were cultured in 96-well culture plates (3000 cells/well) and incubated overnight before treatment. They were divided into 3 groups: control (cell culture medium with 1% FBS), IgA1-MES-treated (appropriate dilution of IgA-MES conditioned medium), Val-treated (appropriate dilution of IgA-MES conditioned medium with 10μM Valsartan) groups. Plates were incubated at 37ºC with 5% CO_2_ for 48 hours. To evaluate the extent of podocyte EMT, Immunocytofluorescence (IF) staining was used to assay the expression levels of desmin, podocalyxin, and podocin.

### 2.7 Screening of Wind-dampness Dispelling TCMs

MPC5 cells were cultured in 96-well plates (3000 cells/well) and incubated overnight before treatment. They were divided into several groups: control (cell culture medium with 1% FBS and without IgA1-MES conditioned medium), IgA1-treated (appropriate dilution of IgA1-MES conditioned medium), Val-treated (appropriate dilution of IgA1-MES conditioned medium and 10μM Valsartan), and TCMs-treated (appropriate dilution of IgA1-MES conditioned medium and monomeric TCMs extract with IC5 concentration) groups. Plates were incubated at 37ºC with 5% CO_2_ for 48 hours.

A total of 21 wind-dampness-dispelling TCM extracts were tested in this study. An equal volume of medium was added to the outermost wells to avoid edge effects in the 96-well plate. Extracts from 21 wind-dampness-dispelling TCMs were screened at the IC5 concentration, which was determined using a CCK8 assay. To evaluate the extent of podocyte EMT, IF staining was used to assay the expression levels of desmin, podocalyxin, and podocin.

### 2.8 Preparation of Extracts for Screening

A total of 250 g of wind-expelling and rheumatism-relieving herbal materials was soaked in water for 4 hours. Subsequently, 2500 mL of water was added, and the mixture was refluxed for 2 hours. The extract was filtered, and the residue was subjected to a second reflux extraction with 2000 mL of water for another 2 hours. After filtration, the extracts were combined and filtered by suction. The resulting solution was concentrated into a paste using a rotary evaporator and then lyophilized for 24 hours to get lyophilized powder. The final product was stored at -20°C for later use.

A 10 mg sample of the lyophilized powder was weighed and dissolved in 1 mL of RPMI-1640 medium to prepare a 10 mg/mL stock solution. After complete dissolution, the solution was filtered through a 0.22-μm low-protein-binding polyether sulfone membrane to sterilize it. The resulting stock solution was stored at -80°C for future use.

### 2.9 Cell Counting Kit 8 (CCK8) Assay

MPC5 cells were plated in 96-well plates at a density of 3000 cells/well and incubated overnight prior to treatment. Extracts from the 21 wind-dampness-dispelling TCMs at different concentrations were added to the wells in the presence of the complete medium for 48 hours. After processing the cells, 10 μL of CCK8 reagent (GlpBio, USA) was added to each well, and the 96-well plate was placed in the incubator for 2 h. The blank group contained only medium and no cells, whereas the control group contained cells but no treatment. Finally, a microplate reader (Varioskan LUX; Thermo Scientific, USA) was used to measure the absorbance (OD) of each well at a wavelength of 450 nm. Cell inhibition rate = (OD control - OD intervention) / (OD control - OD blank).

### 2.10 ELISA of TNF-α in Culture Supernatants

Sv40 MES13 cells were stimulated with different concentrations of pIgA (25, 50, 100, and 150 µg/ml) for different durations (24 and 48 hours). The supernatant was collected and stored at -70°C for the TNF-α assay. The concentration of TNF-α in cell culture supernatants was quantitated according to the manufacturer’s instructions of the mouse TNF-α ELISA kit (ELK Biotechnology, China).

### 2.11 IF Staining

Cells (SV40 MES13 or MPC5) were fixed with 4% paraformaldehyde, permeabilized with 1% Triton X-100, and blocked with goat serum for 60 min, followed by incubation with primary antibodies overnight at 4 °C. The primary antibodies used included: α-SMA antibody (1:200, Immunoway, China), desmin antibody (1:200, Santa Cruz, CA, USA), podocin antibody (1:200, Santa Cruz), and podocalyxin antibody (1:200, CUSABIO, China).

The next day, the cells were incubated with secondary antibodies in the dark for 60 min at 37 °C. The following secondary antibodies were used: goat anti-mouse IgM mu chain - Alexa Fluor 555 (1:1000; Immunoway, China), goat anti-mouse IgG (H&L) - Alexa Fluor 555 (1:1000; Immunoway, China), and goat anti-mouse IgG (H&L) - Alexa Fluor 488 (1:1000; Immunoway, China). Finally, cells were incubated with Hoechst 33342 for 5 min to stain the nuclei.

### 2.12 Image Acquisition and Processing

Fluorescent labeling with AF488 was used for α-SMA staining, whereas AF555 was used for desmin, podocalyxin, and podocin staining. Fifty images per well were captured using an Operetta CLS system equipped with a 20x objective lens. To detect the marker protein labeled green by AF488, Channel 1 was excited at 460 nm. Channel 2 was excited at 540 nm to detect the nuclei stained yellow by AF555. Finally, Channel 3 was excited at 346 nm to visualize nuclei stained blue with Hoechst 33342. Image analysis was performed using the Harmony software (PerkinElmer, Waltham, MA, USA).

### 2.13 Statistical Analysis

All experiments were conducted in triplicate. The results are presented as the mean ± Standard Deviation (SD) for continuous normally distributed data. Statistical analyses were performed using the Statistical Package for Social Science (SPSS) Software version 26.0, with a statistical significance threshold set at *P* < 0.05. Group comparisons were assessed using a one-way Analysis of Variance (ANOVA) or Student's t-test.

## 3 RESULTS

### 3.1 Development of the HCS Assay for Screening Wind-dampness Dispelling TCMs with Anti-EMT Effects

**Fig. (1):**
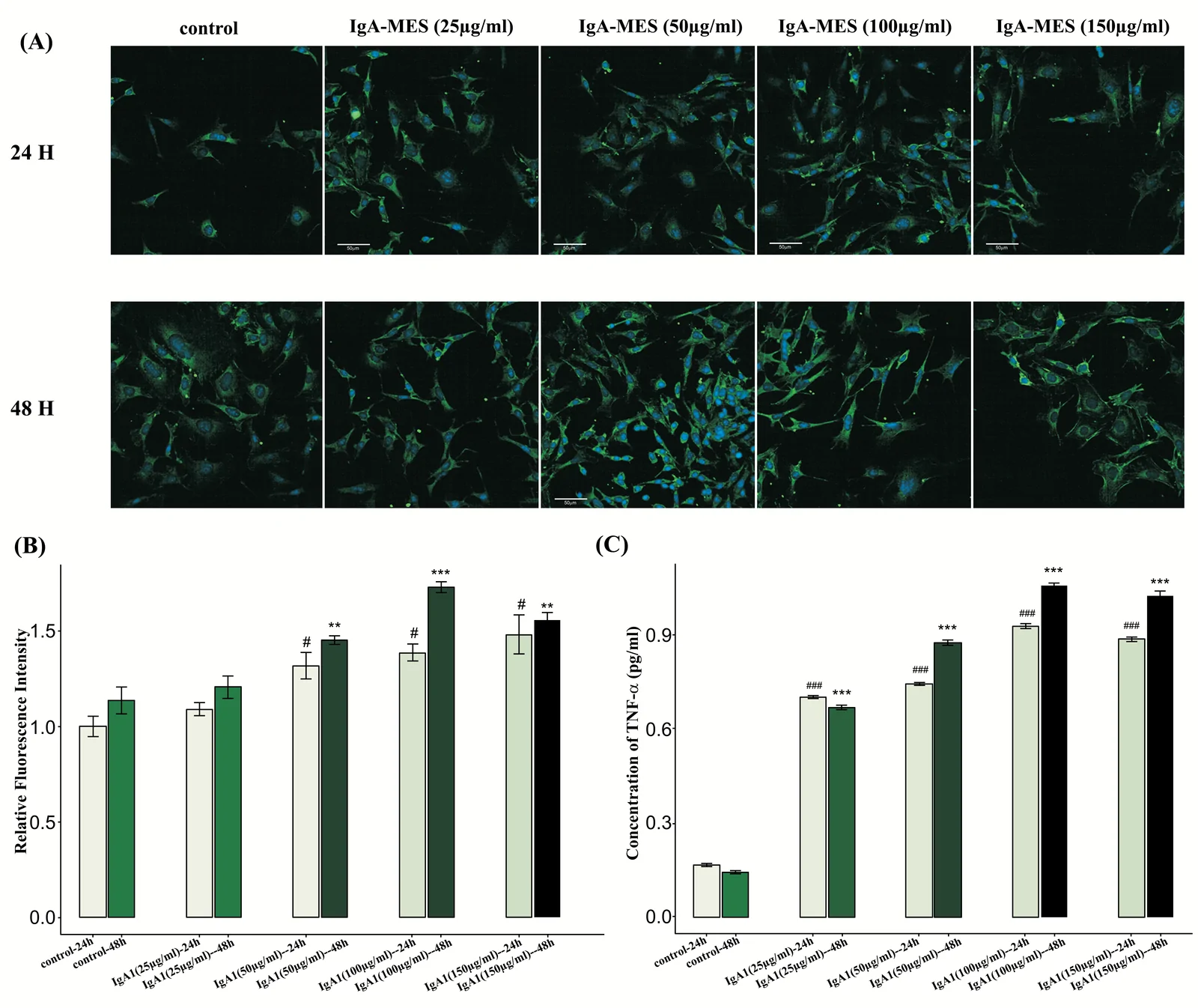
Optimization of mesangial cell stimulation conditions for IgA1. SV40 MES13 cells were subjected to stimulation with varying concentrations of pIgA1 (25, 50, 100, or 150 μg/ml) or untreated (control) for either 24 or 48 hours. (**A**) Representative images(20X) display stained SV40 MES13 cells, with α-SMA stained using AF488 (in green) and cell nuclei stained with Hoechst 33342 (in blue). (**B**) Quantification of changes in α-SMA mean fluorescence intensity in SV40 MES13 cells. (**C**) Measurement of TNF-α concentration in the culture supernatant.

During IgA1 stimulation of mesangial cells, the concentration of TNF-α in the culture supernatant of SV40 MES13 cells was positively correlated with the concentration of IgA1 in a time-dependent manner. After stimulation of IgA1(100ug/ml) for 24 or 48 hours, the 25–150 μg/ml IgA1 group had a statistically significant effect on the concentration of TNF-α (*P* < 0.05), whereas the concentration of TNF-α increased most with the 100 μg/ml IgA1 treated SV40 MES13 cells for 48 h (Fig. **1A**). The fluorescent images in Fig. (**1B**) show that IgA1 at the 100 μg/mL concentration resulted in prominent fluorescence intensity of α-SMA expression after incubation for 48 hours. Based on the above results, the optimal stimulatory IgA1 concentration was determined to be 100 μg/ml, and the optimal stimulation time was 48 hours (Fig. **1C**).

IgA-MES from patients with IgAN upregulated the podocyte expression of desmin and downregulated the podocyte expression of podocalyxin and podocin. Valsartan was used as the reference compound to verify the accuracy and reliability of the HCS system.

**Fig. (2):**
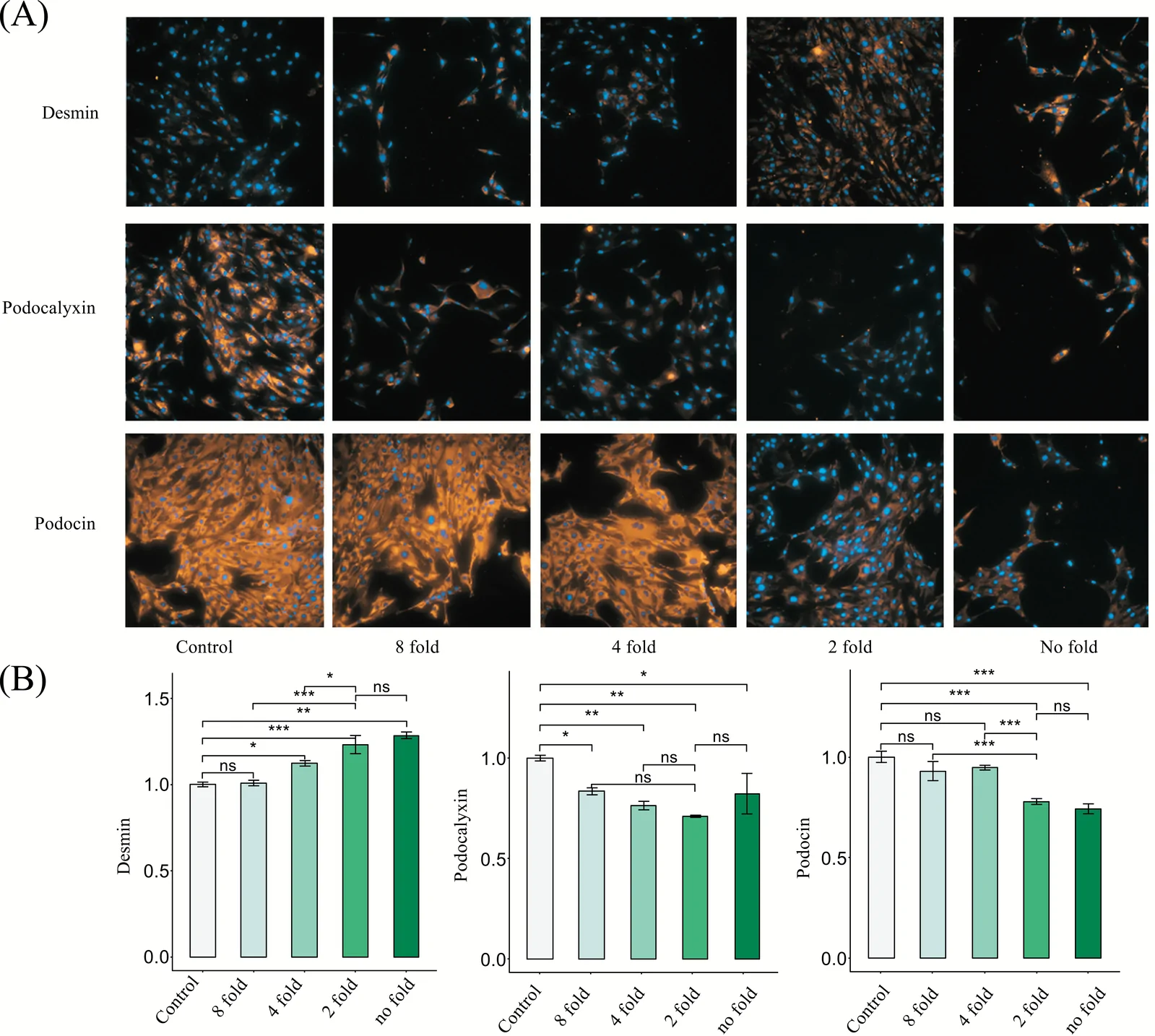
Expression of EMT-related proteins in podocytes after exposure to different concentrations of IgA1-MES. MPCs were exposed to IgA-MES-conditioned medium with varying levels of stimulation, including no stimulation, 2-fold, 4-fold, and 8-fold stimulation, or untreated (control) for 48 hours. (**A**) Representative stained images(20X) of MPCs following the different stimulation conditions (no stimulation, 2-fold, 4-fold, 8-fold, or control) over 48 hours (*n* = 3). (**B**) The protein expressions of podocalyxin and podocin exhibited a significant decrease in podocytes after incubation with different doses (ranging from undiluted to 2-fold diluted medium) of the IgA–MES-conditioned medium derived from IgAN, whereas the protein expressions of desmin increased compared to that from podocytes cultured with the control medium.

Compared to the control group, the expression of podocalyxin and podocin decreased significantly (*P* < 0.05), and the expression of desmin increased significantly (*P* < 0.05) when the stimulation conditions of IgA1-MES treated podocytes were not diluted or doubled. Although protein expression in the non-diluted group changed, there was no significant difference (*P* > 0.05) when compared to the 2-fold diluted group (Fig. **2**). Therefore, a 2-fold dilution was selected as the model condition.

**Fig. (3):**
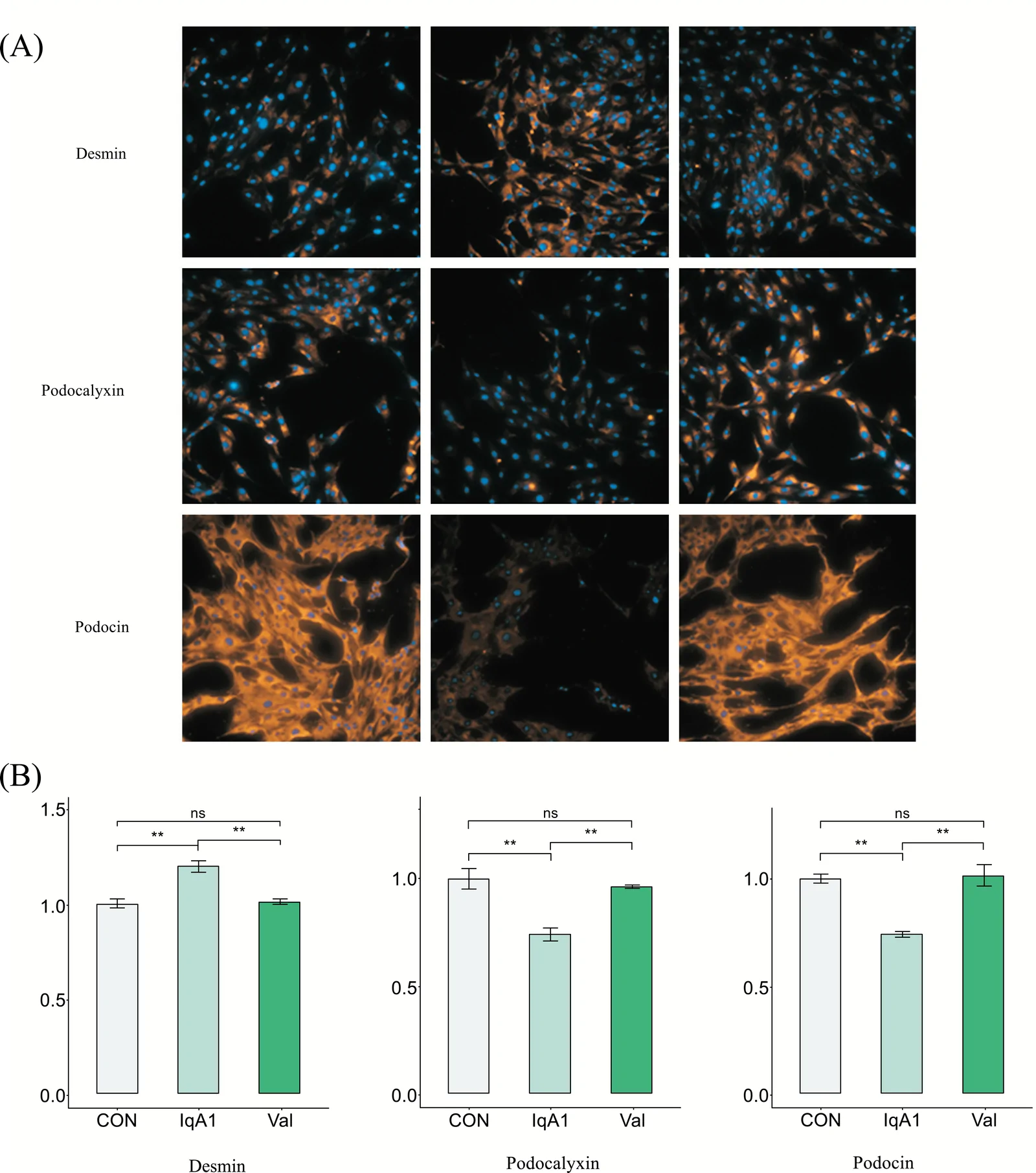
Expression of desmin, podocalyxin, and podocin in podocytes cultured under different conditions. (**A**) Representative stained images(20X) of MPCs following the different stimulation groups over 48 hours (*n* = 3). (**B**) The mean fluorescence intensity of desmin, podocalyxin, and podocin was compared with that from podocytes cultured with the 2-fold IgA-MES conditioned medium.

Compared to the IgA1 group, the expression of podocalyxin and podocin in the Valsartan group was increased, and the expression of desmin was downregulated (*P* < 0.05). There were no significant differences (*P* > 0.05) in the expression of desmin, podocalyxin, or podocin between the Valsartan group and the control group (Fig. **3**).

These findings suggested that a method for quantifying the expression of desmin, podocalyxin, and podocin during EMT based on the HCS system was established. The established HCS system was accurate and reliable.

### 3.2 Validation of Anti-EMT Activity of Wind-dampness Dispelling TCMs

**Fig. (4):**
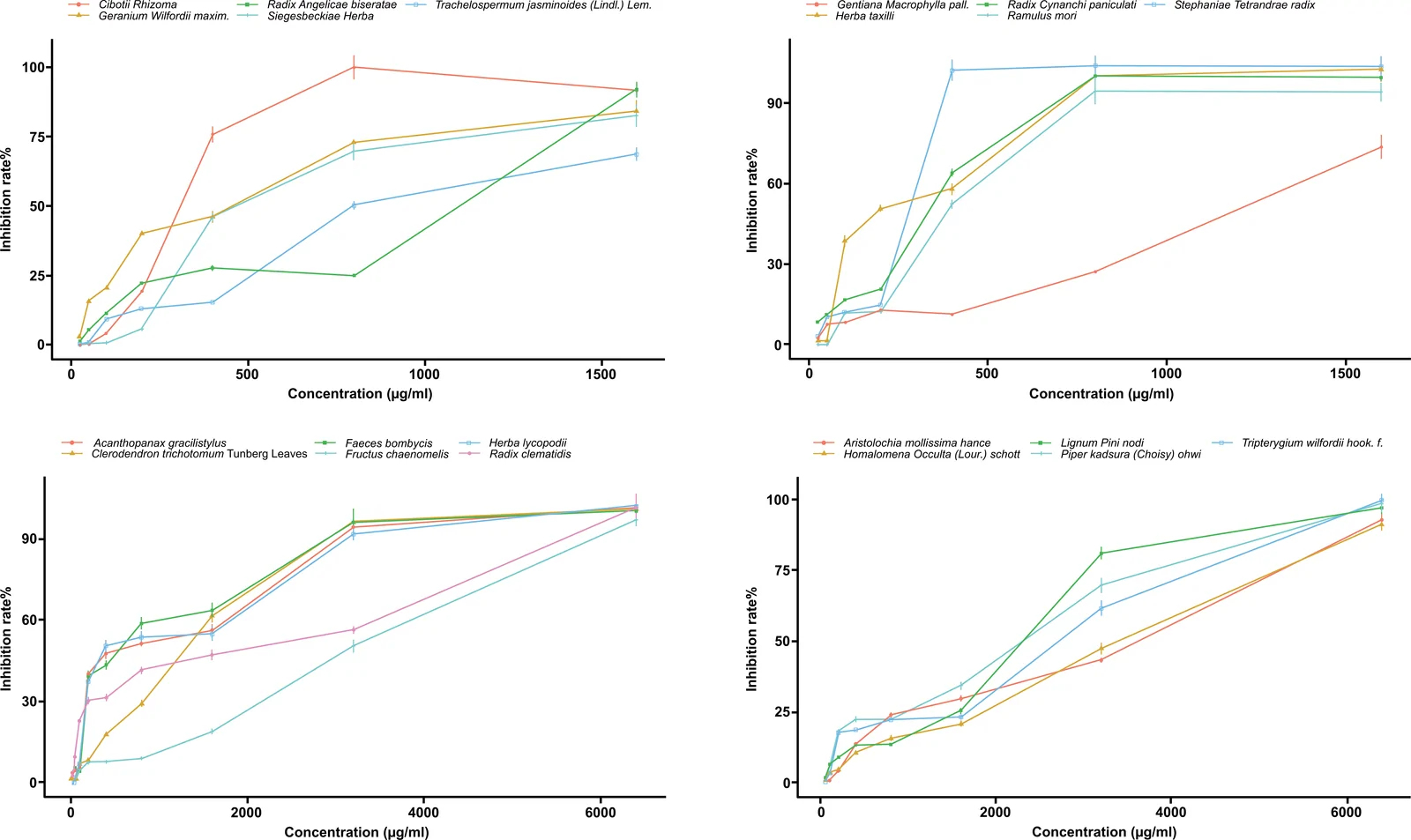
Inhibition rates of extracts from 21 wind-dampness dispelling TCMs on MPC5 cells vitality. MPC5 cells were stimulated with different concentrations of extracts from 21 wind-dampness-dispelling TCMs for 48 hours.

After exposure to different TCM concentrations for 48 hours, a Cell Counting Kit 8 (CCK8) assay was used to determine the optimal concentration of TCM for stimulating MPC5 cells (Fig. **4**). IC5 was used as the intervention concentration (Table **1**).

**Fig. (5):**
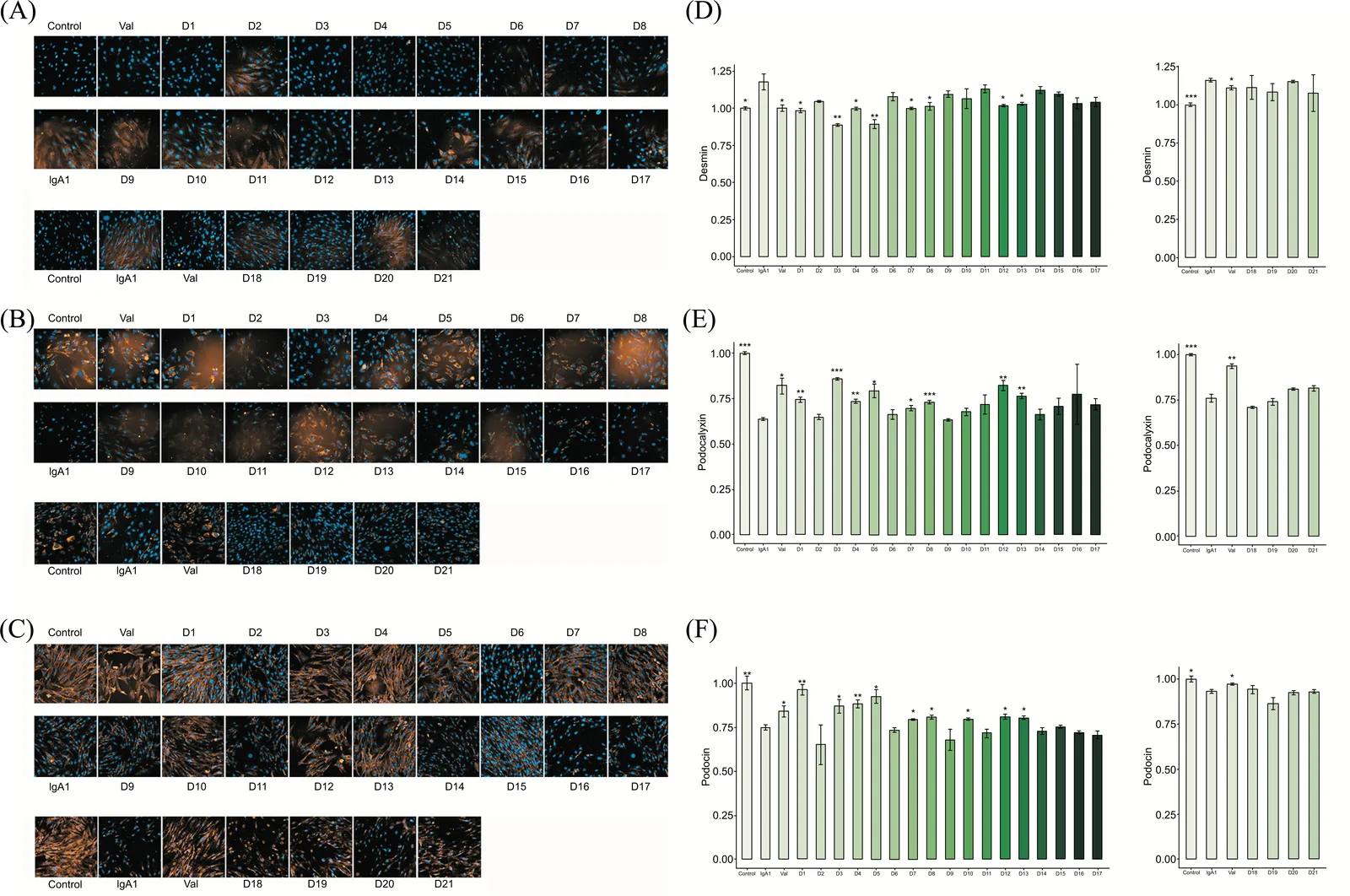
Effects of extracts from 21 wind-dampness dispelling TCMs on MPC5 Expression of EMT-Related Proteins. MPC5 cells were stimulated with extracts from 21 wind-dampness dispelling TCMs, IgA1-MES, Valsartan, or untreated (control) for 48 hours (*n* = 3). Representative stained images(20X) of desmin (**A**), podocalyxin (**B**), and podocin (**C**), along with the mean fluorescence intensity of desmin (**D**), podocalyxin (**E**), and podocin (**F**) compared with data from podocytes cultured with the control medium.

We examined the expression of desmin, podocalyxin, and podocin in podocytes after various stimulations. Alexa Fluor 555 goat anti-mouse IgG (H + L) (1:200) was used to detect desmin, podocalyxin, and podocin. After stimulation with IgA-MES medium in the absence of TCMs, desmin expression was increased compared to that in the control group, whereas podocalyxin and podocin expression were down-regulated (Fig. **5**). Compared to the IgA1 treatment group, the expression of podocalyxin and podocin was significantly increased, and that of desmin was significantly decreased in the groups treated with *Stephaniae Tetrandrae Radix, Trachelospermumjasminoides, Radix Cynanchi Paniculati, Radix Angelicae Biseratae, Radix Clematidis, Caulis Piperis Kadsurae,* and *Tripterygii Radi*x (P<0.05). This suggests that these eight wind-dampness-dispelling TCMs may counteract IgA1 effects and play a role in inhibiting or reversing EMT.

## 4 DISCUSSION

IgAN is characterized by podocyte injury, leading to proteinuria and declining kidney function, which play crucial roles in the progression to End-Stage Renal Disease (ESRD). Unlike primary glomerular diseases, such as minimal change disease and Focal Segmental Glomerulosclerosis (FSGS), podocyte injury in IgAN is predominantly influenced by cellular interactions and inflammation-mediated pathological damage. Previous studies have indicated that abnormal glycosylation of IgA1 deposits in the mesangial and para-mesangial regions of glomeruli can induce podocyte apoptosis in IgAN, thus significantly contributing to proteinuria in IgAN [10]. TNF-α is an important inflammatory factor with a concentration-dependent relationship with podocyte injury. α-SMA is the major marker of mesangial cell sclerosis. Previous studies have speculated that the proliferation of mesangial cells and the release of inflammatory factors cause podocyte injury [11]. EMT is an initial and major process resulting in renal abnormalities, including podocyte apoptosis [12]. During this process, the expression of podocyte-specific markers such as podocalyxin and podocin is inhibited, whereas that of mesenchymal markers such as desmin is elevated [13, 4].

According to traditional Chinese medicine theory, IgAN patients were classified into Qi yin deficiency, blood stasis in the kidney, and wind-dampness interfering in the kidney [7]. Wind-dampness interfering in the kidney is not only the primary syndrome in patients with massive proteinuria but is also associated with severe podocyte damage and an increased risk of renal function progression. The dispelling of wind-dampness is an effective approach for treating IgAN, as it can significantly reduce proteinuria, protect renal function, and improve prognosis [7, 14, 15].

However, many wind-dampness drugs exist, and most of them lack experimental validation. Therefore, our focus is to identify effective wind-dampness drugs and explore their mechanisms of action.

The goal of this study was to screen for wind-dampness-dispelling TCM that could inhibit podocyte EMT induced by IgA1-stimulated mesangial cell culture supernatants.

Previous studies have demonstrated that pIgA from patients with IgAN does not activate podocytes directly and that podocytes lack a known IgA receptor [16]. Therefore, we conducted podocyte culture experiments using a conditioned medium from mesangial cells pre-incubated with the IgA1 preparation. We detected the TNF-α concentration in the supernatant and the expression of α-SMA in mesangial cells to determine the induction of IgA1 mesangial cell conditions. Thus, we focused on these markers to assess the extent of EMT. The optimal conditions for IgA1-MES-induced EMT in MPC5 cells were determined, and the screening method was validated using Valsartan. The *in vitro* model established using this method exhibited good stability and high reproducibility. It can be used for HCS screening of antirheumatic drugs.

In our study, a total of 21 wind-dampness dispelling TCMs, including Stephaniae Tetrandrae Radix, Gentiana Macrophylla Pall., Trachelospermum jasminoides (Lindl.) Lem., Geranium Wilfordii Maxim, Radix Cynanchi Paniculati, Fructus Chaenomelis, Radix Angelicae Biseratae, Radix Clematidis, Lignum pini nodi, Herba Taxilli, Aristolochia mollissima Hance, Piper kadsura (Choisy) Ohwi, Tripterygium wilfordii Hook. f., Siegesbeckiae Herba, Cibotii Rhizoma, Ramulus Mori, Acanthopanax gracilistylus, Faeces Bombycis, Herba Lycopodii, Homalomena Occulta (Lour.) Schott, and Clerodendron trichotomum Tunberg Leaves were screened. Our results showed that the extracts of Stephaniae Tetrandrae Radix, Trachelospermum jasminoides (Lindl.) Lem., Radix Cynanchi Paniculati, Radix Angelicae Biseratae, Radix Clematidis, Caulis Piperis Kadsurae, and Tripterygium wilfordii Hook. f. decreased desmin expression and increased expression of podocalyxin and podocin, inhibiting IgA1-MES-induced EMT in MPC5 cells.

*Tripterygium wilfordii Hook. f.* has been reported to inhibit EMT, reduce proteinuria, and exert renal protective effects [8, 17-21]. Studies have found that hesperetin, one of the active ingredients of *Stephaniae Tetrandrae Radix*, can inhibit EMT in tumor cells by regulating the mTOR pathway [22]. Tetrandrine, the main active component isolated from the Chinese herb *Stephaniae Tetrandrae Radix*, was confirmed to alleviate podocyte injury induced by TRPC6 expression through the inhibition of the RhoA/ROCK pathway, suggesting a protective role in podocyte damage [23]. It is found in *Trachelospermum jasminoides* (Lindl.) Lem., a traditional herbal remedy that reduces proteinuria and has been suggested to have a renoprotective mechanism *via* PP2A activation [24].

Identification of the inhibitory actions of Radix Cynanchi Paniculati, Radix Angelicae Biseratae, Radix Clematidis, Piper kadsura (Choisy) Ohwi, and Geranium wilfordii Maxim. on IgA1-MES-induced EMT in podocytes is novel.

## CONCLUSION

In conclusion, we established a rapid, reliable, and high-throughput screening method based on the HCS system to assess podocyte EMT. Eight wind-dampness dispelling TCMs were screened from 21 candidates and were shown to have anti-EMT activity.

## STUDY LIMITATIONS

Although these findings will stimulate interest in the potential use of wind-dampness-dispelling TCMs for the treatment of kidney diseases, we acknowledge the limitations of this study. This research is solely based on *in vitro* models. Although we have identified potentially effective wind-dampness drugs *in vitro*, their specific mechanisms have not been thoroughly explored. Therefore, the guiding value of this study for clinical applications is limited. Our team is conducting follow-up research on the underlying mechanisms to provide a stronger foundation for clinical practice.

## Figures and Tables

**Table 1:** IC5 and IC50 of extracts from 21 wind-dampness dispelling TCMs determined using the CCK8 assay.

**DURGs**	**IC5**	**95%CI**	**IC50**	**95%CI**	**Conceration**
*Stephaniae Tetrandrae Radix*	45.21	0-115.2	199.6	45.5-936.6	50ug/ml
*Gentiana Macrophylla Pall.*	67.67	7.2-154.7	1574.8	731.8-11152.3	70ug/ml
*Trachelospermum jasminoides (Lindl.) Lem.*	113.88	42.4-190.5	971.3	659.4-1778	120ug/ml
*Geranium Wilfordii Maxim*	26.44	16.6-37.3	367.5	301.4-465.6	30ug/ml
*Radix Cynanchi Paniculati*	33.65	4.7-73.8	245.1	130.3-495.2	30ug/ml
*Fructus Chaenomelis*	319.2	18.6-733	2725.9	1385.8-10128.6	300ug/ml
*Radix Angelicae Biseratae*	70.97	1.7-177.9	796.7	379.4-5079.8	70ug/ml
*Radix Clematidis*	27.05	2.9-76.6	1103.1	581.7-2641.4	30ug/ml
*Lignum pini nodi*	209.05	21.3-473.5	1792.2	942.3-4514.8	200ug/ml
*Herba Taxilli*	42.51	11.4-78.1	219.4	136.7-354.2	50ug/ml
*Aristolochia mollissima Hance*	263.57	106.6-445	2510.2	1739.8-4078.3	250ug/ml
*Piper kadsura (Choisy) Ohwi*	130.04	35.8-257.4	1616.1	1006.1-2980.2	130ug/ml
*Tripterygium wilfordii Hook. f.*	158.61	12.7-388.5	1923.6	974-5741.7	150ug/ml
*Siegesbeckiae Herba*	147.33	66.7-224.6	616.2	457.4-871.4	150ug/ml
*Cibotii Rhizoma*	98.59	20.8-172.9	340.4	204.6-575.5	100ug/ml
*Ramulus Mori*	96.17	41.4-151	403.5	290.6-572.1	100ug/ml
*Acanthopanax gracilistylus*	45.5	11.8-95.9	630.2	384.6-1071.8	50ug/ml
*Faeces Bombycis*	49.49	18.6-91	598.2	405.1-899.3	50ug/ml
*Herba Lycopodii*	60.14	14.2-127.3	660.7	389.8-1165.3	60ug/ml
*Homalomena Occulta (Lour.) Schott*	311.66	89.7-573.3	2844.4	1816.7-5552.1	300ug/ml
*Clerodendron trichotomum Tunberg Leaves*	152.27	60.5-260.1	1031	710.3-1536.1	150ug/ml

## Data Availability

The data and supportive information are available within the article.

## LIST OF ABBREVIATIONS

EMT: Epithelial-to-Mesenchymal Transition
HCS: High-Content Screening
IF: Immunocytofluorescence
IgA: Immunoglobulin A
IgAN: Nephropathy
TCMs: Traditional Chinese Medicines

## STANDARDS OF REPORTING

STROBE guidelines were followed.
